# Effects of basin-scale climate modes and upwelling on nearshore marine heatwaves and cold spells in the California Current

**DOI:** 10.1038/s41598-023-39193-4

**Published:** 2023-07-31

**Authors:** Michael Dalsin, Ryan K. Walter, Piero L. F. Mazzini

**Affiliations:** 1 Physics Department, California Polytechnic State University, San Luis Obispo, CA USA; 2 Virginia Institute of Marine Science, William & Mary, Gloucester Point, VA USA

**Keywords:** Physical oceanography, Physical oceanography

## Abstract

Marine heatwaves and cold spells (MHWs/MCSs) have been observed to be increasing globally in frequency and intensity based on satellite remote sensing and continue to pose a major threat to marine ecosystems worldwide. Despite this, there are limited in-situ based observational studies in the very shallow nearshore region, particularly in Eastern Boundary Current Upwelling Systems (EBUS). We analyzed a unique dataset collected in shallow waters along central California spanning more than four decades (1978–2020) and assessed links with basin-scale climate modes [Pacific Decadal Oscillation (PDO) and El Niño (MEI)] and regional-scale wind-driven upwelling. We found no significant increase/decrease in MHW/MCS frequency, duration, or intensity over the last four decades, but did observe considerable interannual variability linked with basin-scale climate modes. Additionally, there was a decrease in both MHW/MCS occurrence during the upwelling season, and the initiation of individual MHWs/MCSs coincided with anomalous upwelling. Most notably, the co-occurrence of warm (cold) phases of the PDO and MEI with negative (positive) upwelling anomalies strongly enhanced the relative frequency of positive (negative) temperature anomalies and MHW (MCS) days. Collectively, both basin-scale variability and upwelling forcing play a key role in predicting extreme events and shaping nearshore resilience in EBUS.

## Introduction

Prolonged extreme sea water temperature anomalies, known as marine heatwaves (MHWs) and marine cold spells (MCSs), can have long-lasting or permanent effects on marine ecosystems. Coral bleaching events, giant kelp forest loss, ecosystem regime shifts, poleward expansion of marine species, mass die-offs of seabirds and economically important fisheries, and harmful algal blooms have all been linked to MHWs^1–4^. MCSs, while understudied, have also been connected with coral bleaching^5^, range shifts in marine species, and large-scale fish mortality events^6^, although they have also been shown to bolster giant kelp forests in Patagonia^7^. MHWs and MCSs affect species across all trophic levels and disrupt food chains, having profound consequences to the marine ecosystem, Blue Economy, and society^8^.

Globally over the last four decades, an increase in the frequency, intensity, and duration of MHWs has been observed, which contrasts with a decrease in the respective quantities for MCSs^6,9,10^. This shift has been attributed to an increase in both the mean and variance of sea surface temperature (SST), coinciding with a surge in greenhouse gas emissions from humans and the resultant warming^11,12^. Deviating from these mean global trends are Eastern Boundary Current Upwelling Systems (EBUS), including the California Current System (CCS), the focus of this study, as well as the Benguela, Canary, and Humboldt currents, which are among the most biologically productive regions in the world^13–15^. Along these regions, no statistically significant changes in MHW frequency, intensity, or duration have been detected based on satellite records of SST dating back to 1982^9^. This has been attributed to wind-driven coastal upwelling, which buffers against MHWs through modulation of SST trends in EBUS^16^. The reduced warming rate and dampened trends in MHWs have led the scientific community to hypothesize that EBUS may serve as thermal refugia and thus play a key ecological role in a warming climate^16–22^.

While satellite remote sensing of SST provides multi-decadal time series that have allowed great advances in MHW/MCS research at global scales, including along EBUS, their applicability to nearshore coastal waters is limited due to known biases and limitations in these regions^16,23,24^. Challenges in SST remote sensing in the coastal zone arise from land run-off, greater SST variability, increased presence of aerosols and water vapor, and inherently coarse resolution that does not adequately resolve coastline features. These remote sensing limitations have hampered MHW and MCS research in coastal regions. To date, only a limited number of studies have been conducted on MHWs using long-term in situ records, particularly in shallow nearshore (< 15 m depth) environments, with limited published work in EBUS^9,21,24–28^. Moreover, we are not aware of any study that has investigated MCSs in the shallow nearshore of EBUS.

Motivated by this lack of understanding of MHWs/MCSs in shallow, nearshore regions in EBUS, which may play a key role in our changing climate, we investigated a novel in-situ temperature record spanning over four decades from a nearshore site along the central California coast. To our knowledge, these data represent the longest in-situ temperature record in an understudied stretch of the central California coast (USA) spanning ~ 300 km between Point Concepcion to the south and Monterey Bay to the north. The goal of this work is threefold: (1) characterize seasonal and interannual variability in MHW and MCS characteristics; (2) analyze potential trends in MHW and MCS characteristics; and (3) assess linkages between MHWs/MCSs with basin-scale climate modes in the Pacific (El Niño Southern Oscillation, Pacific Decadal Oscillation) and regional-scale coastal upwelling to develop statistical likelihoods for nearshore extreme events. This work provides the first assessment of both MHWs/MCSs in the shallow nearshore location in an EBUS and can be used as a framework for other sites across the CCS and in other EBUS globally.

## Results

### General observations

**Figure 1:**
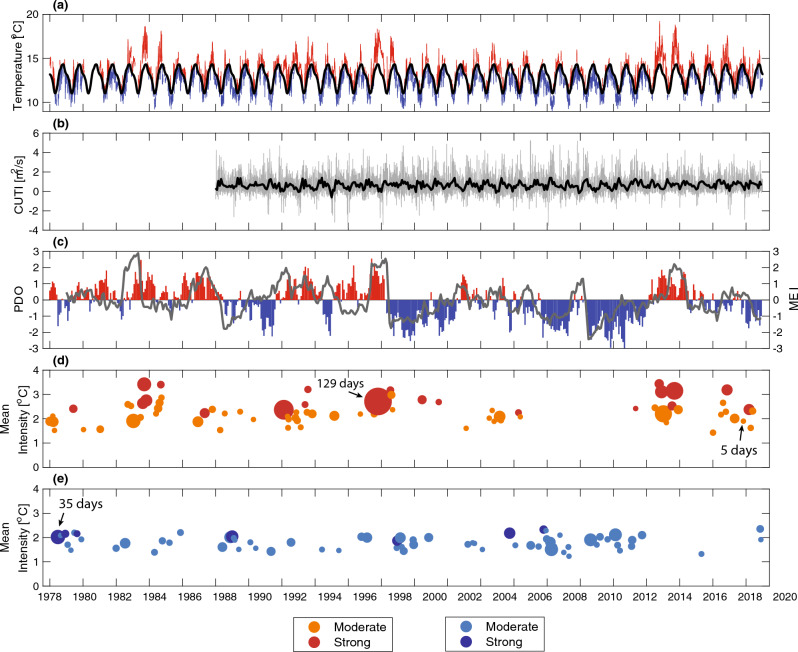
Time series from 1978 to 2020 of the (**a**) daily averaged temperature where red (blue) indicates positive (negative) anomalies from the calculated climatology (black), (**b**) daily averaged (gray) and monthly averaged (black) CUTI (only available from 1988 onward), (**c**) monthly PDO climate index (left axis) showing positive/warm phase (red bars) and negative/cold phase (blue bars) and the monthly MEI (right axis; solid gray line), (**d**) mean intensity of MHWs, and (**e**) mean intensity of MCSs. The size in panels (**d**) and (**e**) is scaled according to the event duration in days, with example labels shown in each panel for scale. For MHWs in panel (**d**), the orange color denotes moderate events and the red color denotes strong events. For the MCSs in panel (**e**), the light blue color denotes moderate events and the dark blue color denotes strong events.

Over the entire time series, 80 MHWs and 69 MCSs were detected, with an average annual frequency of 1.86 and 1.61 events per year for MHWs and MCSs, respectively. The majority of events were classified as moderate (58 for MHWs, 63 for MCSs), with 30% of MHWs and 11% of MCSs classified as strong (Table 1). MHWs had a mean duration of 13.80 days and an average event-mean intensity of 2.3 °C. MCSs had a mean duration of 10.72 days and an average event-mean intensity of 1.8 °C (Table 1). The maximum event-mean intensity was 3.4 °C during a MHW in 2014 and 2.4 °C during a MCS in 2020. The longest MHW was 129 days (1997–1998, coinciding with a major El Niño event) while the longest MCS was 35 days (1978). On average, MHWs were more prolonged, more intense, and more frequent than MCSs. For the metrics reported, across all events the variability (as measured by the standard deviation), was larger for MHWs compared to MCS (see “All Events” rows in Table 1). The most intense and longest duration MHWs typically occurred during warm phases of the PDO and during the largest El Niño (positive MEI) events (e.g., 1983–1984, 1992, 1997–1998, 2015; Fig. 1). On average, there were 25.7 MHW days per year and 17.2 MCS days per year. The average annual cumulative intensity was 63.3 °C × days/year for MHWs and 31.9 °C × days/year for MCSs (Table 1). No linear trends in MHW or MCS metrics were found to be statistically different from zero at the 95% confidence level.

**Table 1:** Summary of MHW and MCS metrics across all events, moderate events, and strong events. The mean and standard deviation (in parentheses) are shown.

Event category	Event type	Number of events	Frequency (events/year)	Duration (days)	Average intensity (°C)	Event days (days/year)	Yearly cumulative intensity (°C × days/year)
All events	MHW	80	1.86 (2.00)	13.80 (17.08)	2.32 (0.48)	25.67 (40.97)	63.29 (107.07)
	MCS	69	1.61 (1.55)	10.72 (7.02)	1.80 (0.26)	17.21 (18.04)	31.85 (33.80)
Moderate events	MHW	55	1.28 (1.44)	9.67 (7.82)	2.11 (0.35)	12.37 (15.83)	26.20 (33.78)
	MCS	62	1.44 (1.42)	9.81 (6.12)	1.78 (0.25)	14.14 (16.34)	25.47 (29.48)
Strong events	MHW	25	0.58 (0.96)	23.12 (26.42)	2.80 (0.38)	13.30 (31.10)	37.09 (86.04)
	MCS	7	0.16 (0.43)	18.86 (9.56)	2.11 (0.15)	3.07 (9.00)	6.38 (18.58)

### Interannual variability and basin-scale drivers

**Figure 2:**
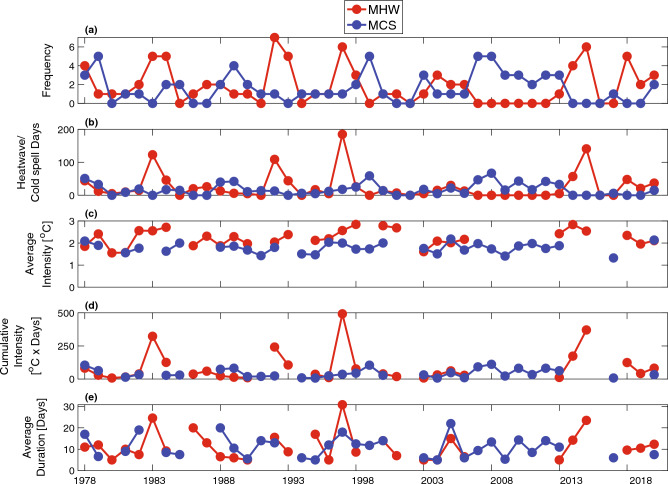
Annual values from 1978 to 2020 of the (**a**) number of events, (**b**) number of event days, (**c**) average event mean intensity magnitude, (**d**) total cumulative intensity magnitude, and (**e**) average event duration. MHWs shown in red and MCSs shown in blue.

Considerable interannual variability was observed in both MHW and MCS metrics (Fig. 2). The occurrence of MHWs and MCSs were inversely related, with intervals of one to seven years of numerous MHWs and no MCSs, and vice versa (Fig. 2a). These patterns closely followed the PDO index, with more MHWs during positive phases and more MCSs during negative phases. During strong El Niño years (large positive MEI; e.g., 1983, 1997, 2015), total MHW days exceeded one hundred days, while other years with MHWs typically had less than approximately fifty total days (Fig. 2b). Cumulative annual MCS days were typically less than fifty days per year with much smaller peaks (e.g., 1999, 2008). The annually averaged event-mean intensity, cumulative intensity, and duration showed similar interannual patterns (Fig. 2c,d).

### Seasonal variability and upwelling

**Figure 3:**
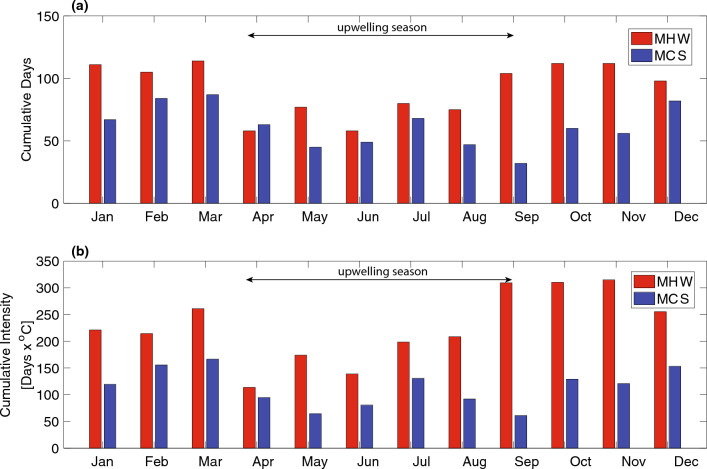
(**a**) Cumulative event days and (**b**) cumulative intensity in each month over the entire time series for MHWs (red) and MCSs (blue).

**Figure 4:**
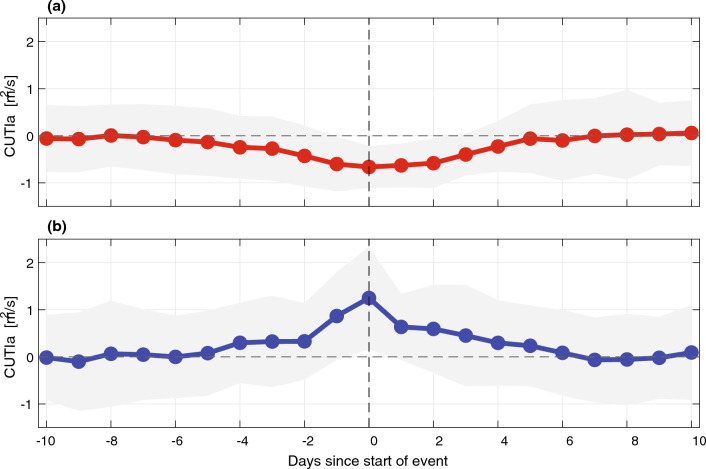
Average CUTI anomaly (CUTIa) before and after the start of (**a**) MHWs and (**b**) MCSs. Solid lines show the mean CUTI anomaly over all events and the light gray shading denotes one standard deviation from the mean.

MHW/MCS days and cumulative intensity showed seasonal variability when binned by month across all years (Fig. 3). For MHWs, September–November contributed the most to total MHW days and intensity, while December-March the most to MCS days and intensity. April-August were the five months that contributed the least to MHWs, coinciding with the major upwelling season in this region^29^. We note that ENSO variability may also be weaker following the boreal winter^30^^.^ Composite averages of the CUTIa (upwelling anomaly) before and after the start of all MHWs and MCSs, respectively, revealed anomalous upwelling conditions linked with the initiation of these events (Fig. 4). Across all MHWs, the composite average CUTIa became increasingly negative several days before the detection of the MHW, with the local minimum observed at the initiation of the event. The negative anomaly observed at the start of the MHW represented either weaker than normal upwelling or downwelling (~ 50% split between these conditions in the dataset). In contrast, the composite average CUTIa across all MCSs became increasingly positive before the detection of the event with a local maximum on the initiation day (Fig. 4). The positive CUTIa corresponded to stronger than normal upwelling since the CUTI climatology was always positive and therefore upwelling-favorable. Approximately five days after the initiation of MHWs and MCSs, the composite average CUTIa returned to values around zero (normal climatological upwelling conditions). These trends in upwelling anomaly indicate that upwelling is important to the initiation of MHWs/MCSs but is not a good predictor of the duration of an event.

### Co-occurrence of basin-scale and regional-scale drivers

**Figure 5:**
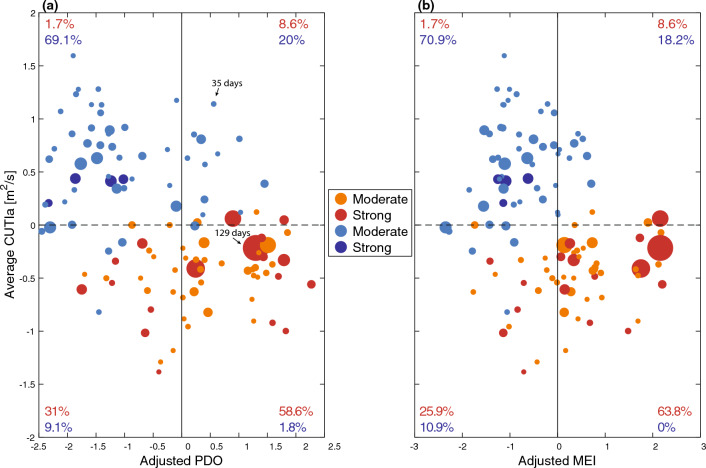
Scatter plots of the average CUTI anomaly vs. the (**a**) adjusted PDO and (**b**) adjusted MEI for all MHWs (orange/red) and MCSs (light/dark blue). Point size scaled by duration and color coded by severity. Percentages shown indicate the proportion of MHW (red) and MCS (blue) events in each respective quadrant for all events.

There was distinct clustering of MHWs and MCSs when examining a basin-scale climate mode (PDO/MEI) in conjunction with the CUTIa (Fig. 5). For the PDO-CUTIa parameter space, 58.6% of the observed MHWs were detected during the co-occurrence of a positive PDO and negative CUTIa, with only 1.8% of the MCSs occurring during these combinations (quadrant 4 of Fig. 5a). In contrast, during a negative PDO and positive CUTIa phase, 1.7% of the MHWs and 69.1% of the MCSs were observed (quadrant 2 of Fig. 5a). A similar pattern was also evident for MEI-CUTIa parameter space (Fig. 5b). Notably, the most prolonged MHWs occurred with the most positive MEI, and MCSs with the most negative PDO.

**Figure 6:**
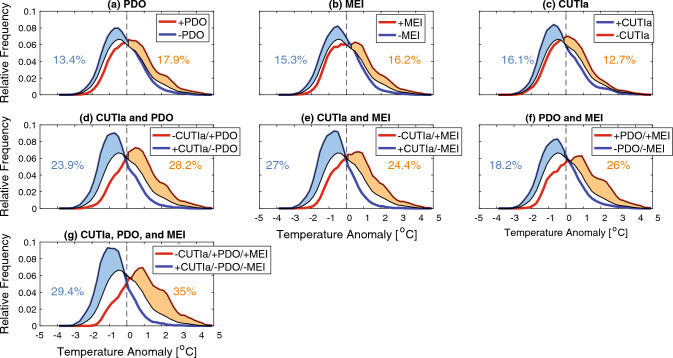
Temperature anomaly histograms with all data 1988–2020 shown in black (same in every panel). In each panel, data were separated using combinations of either the PDO, MEI, and/or CUTIa. All blue colors denote negative PDO, negative MEI, and/or positive CUTIa, while all red colors denote positive PDO, positive MEI, and/or negative CUTIa. The orange (light blue) shading represents increased relative frequency of warm/positive (cold/negative) temperature anomalies during red (blue) combinations relative to all data.

The frequency distribution of temperature anomalies across the entire dataset peaks at approximately − 0.5 °C but is overall skewed in the positive direction (longer positive tail; Fig. 6). Analysis of temperature anomaly histograms sorted by the phase of an individual oceanic index (PDO, MEI, or CUTIa) highlighted that individual drivers resulted in increased frequency of temperature anomalies. For example, a positive PDO phase resulted in a 17.9% increase in warm temperature anomalies relative to the entire dataset with the peak and tail both shifted in the positive direction (orange shaded region in Fig. 6a). The co-occurrence of multiple drivers produced even larger differences in the anomaly histograms (Fig. 6d–g). The largest difference occurred when including all three indices (PDO/MEI/CUTIa). There were 35.0% more warm temperature anomalies relative to the entire dataset with the combination of a positive PDO, positive MEI, and negative CUTIa. There were 29.4% more cold temperature anomalies for the combination with the opposite signs (Fig. 6g).

**Figure 7:**
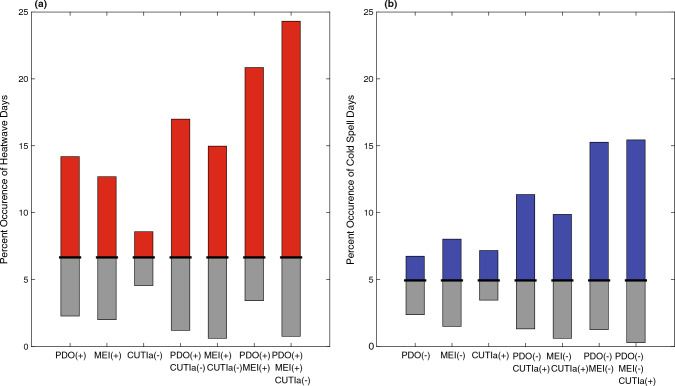
Percent occurrence of (**a**) MHW and (**b**) MCS days during different combinations of the PDO, MEI, and/or CUTIa (as in Fig. 6). Red and blue bars represent increased occurrence of MHWs and MCSs, respectively, in the different combinations, while gray bars represent decreased occurrence, both relative to the occurrence across the entire data set (black bars).

To quantify the influence of the different oceanic indices on the occurrence of MHWs/MCSs, we calculated the percent of days classified as a MHW or MCS over the entire dataset and compared this to the percent of days classified as a MHW or MCS when sorting the data by the sign of different parameters and their combinations (Fig. 7). The change in percent occurrence of MHWs/MCSs from the full dataset to the sorted data represents an increase or decrease in the likelihood of MHWs/MCSs during different parameter combinations. For example, percent occurrence for MHW days over the entire dataset was 6.7%. When sorting the data by the sign of the PDO, the percent occurrence changed to 14.2% for the positive phase and 2.3% for the negative phase. For individual drivers (PDO, MEI or CUTIa) the percent occurrence did not exceed 15% for MHWs and 9% for MCSs. The co-occurrence of multiple forcing parameters greatly enhanced the percent occurrence change of MHWs/MCSs. Co-occurrence of all three oceanic indices led to 24.3% occurrence of MHWs (positive PDO and MEI, negative CUTIa) and 15.4% occurrence of MCSs (negative PDO and MEI, positive CUTIa) with the opposite combinations having 0.8% and 0.3% occurrence for MHWs and MCSs, respectively.

## Discussion and conclusions

### Trends and EBUS as thermal Refugia

Globally, few studies have been conducted on MHWs or MCSs in very shallow nearshore regions), with most of those studies relying on satellite-based measurements, which have numerous problems when assessing nearshore sea surface temperatures, especially in EBUS^24,26–28^. To the best of our knowledge this is the first nearshore analysis of both MHWs and MCSs along with their potential drivers using in-situ measurements in the CCS. We found no significant increase in MHW frequency or intensity nor decrease in the same MCS quantities over the last four decades. This is in contrast to global trends elucidated by both satellite and in-situ measurements in other regions and boundary currents^9,10,26^. However, our findings align with previous in-situ studies from the CCS that found very subtle trends in MHWs (MCSs were not analyzed) only observable on time scales greater than four decades^9^. Furthermore, these findings support the hypothesis that EBUS may serve as future thermal refugia based on observations that these ecologically productive regions have generally experienced less warming compared with other regions in recent decades^20^. The lack of significant trends in MHW/MCS metrics at our site are consistent with other EBUS studies that found much slower rates of warming in the nearshore compared to offshore^17–19^.

### Co-occurrence of basin-scale and regional-scale drivers

Basin-scale (PDO and MEI) and regional-scale (upwelling/CUTI) forcing parameters were correlated with temperature anomalies and consequently with the relative frequency of MHWs and MCSs. While individually these parameters moderately affected the relative frequency of MHWs and MCSs, the co-occurrence of multiple parameters strongly enhanced both positive/negative temperature anomalies and MHW/MCS days (Figs. 6, 7). Dynamically, these processes operate on distinct timescales with basin-scale climate modes driving low-frequency (interannual) variability in the background state and positive phases associated with increased SST, thermocline depth, and upper ocean stratification^31,32^. Upwelling drives higher-frequency fluctuations in temperature and stratification in this region both seasonally and intraseasonally (synoptically) due to one to two week upwelling/relaxation cycles^29,33–36^. Given the time-scales of MHWs/MCSs (days to months; see Table 1 and Fig. 1d,e), for all but the longest duration events, the low-frequency background state of the upper ocean set by basin-scale climate modes (e.g., PDO/MEI) changes minimally across a MHW/MCS, whereas upwelling anomalies (CUTIa) can be highly variable. Thus, when examining the percent occurrence of MHW and MCS days for a given sign of these parameters, there is a much stronger association between MHWs and MCSs with the PDO and MEI compared to the CUTIa (Fig. 7). This is also evident in the temperature anomaly histograms (Fig. 6). During warm phases of the PDO and MEI (MHW-favorable), there is both a greater increase in warm temperature anomalies and a larger proportion of anomalies occurring in the higher temperature region compared to periods of negative CUTIa (also MHW-favorable; compare Fig. 6a,b with Fig. 6c above 2 °C). Given that the most extreme warm temperature anomalies were more correlated with basin-scale climate modes, it is not surprising that the longest duration MHWs occurred during strong PDO and MEI events that persisted for months and longer (Figs. 1, 6). The combined influence of a positive PDO and MEI for the occurrence of extreme warm water anomalies along the US West Coast is consistent with previous findings^37,38^.

On shorter time scales, upwelling anomalies play a key role in driving MHWs and MCSs by modifying the low-frequency background temperature and stratification, a finding that likely extends to other EBUS. In addition to the seasonal trend in MHWs and MCSs associated with upwelling seasonality (Fig. 3), there is a clear link between the initiation of MHWs and MCSs with anomalous upwelling (positive CUTIa = strong upwelling linked with MCS initiation; negative CUTIa = weak upwelling/downwelling linked with MHW initiation; Fig. 4). These upwelling anomalies tend to be short-lived with composite CUTIa averages returning close to zero approximately five days after the initiation of both MHWs and MCSs (Fig. 4). Even so, this anomalous upwelling at the initiation of MHWs and MCSs led to non-zero average CUTIa values across each event duration (e.g., clustering of MHWs in positive CUTIa quadrants and MCSs in negative CUTIa quadrants; Fig. 5).

We hypothesize that upwelling anomalies can initiate and sustain MHWs and MCSs by driving changes to background temperature and stratification through modification of the depth of the upwelling-induced cross-shelf return flow. This is best illustrated using the slope Burger number, $S= \alpha N/f$, where $\alpha$ is the topographic slope of the adjacent shelf, *N* is the buoyancy frequency and a measure of stratification, and *f* is the Coriolis frequency^39,40^. Smaller Burger numbers, driven by less stratification for a fixed topographic slope and Coriolis frequency, yield a cross-shelf return flow concentrated in the bottom boundary layer such that colder waters are fluxed into the nearshore during upwelling. On the other hand, larger Burger numbers, due to increases in stratification, result in cross-shelf return flows concentrated in the middle of the water column such that less cold waters are transported into the nearshore. For example, MHW initiation was linked with a negative CUTIa (weaker upwelling), which is expected to increase stratification, leading to a larger Burger number. This leads to an interior return flow that diminishes the effectiveness of upwelling in providing a cold-water reprieve in the nearshore. Similarly, MCS initiation was linked with a positive CUTIa whereby stronger upwelling anomalies lead to less stratification, a smaller slope Burger number, and bottom-concentrated return flows with colder waters transported into the nearshore. In both cases, once the upwelling anomaly subsides, the MHW/MCS can still persist given the location of the return flow and slowly changing background state set by basin-scale climate modes. This slope Burger number feedback mechanism potentially explains why the sign of the CUTIa is linked with initiation of MHWs and MCSs but not with duration, as well as why the co-occurrence of upwelling anomalies and basin-scale climate modes displayed the greatest skill in predicting the percent occurrence of MHW/MCS days (e.g., warm/positive phase of PDO and MEI, with a negative CUTIa for MHWs; cold/negative phase of PDO and MEI, with a positive CUTIa for MCSs; Fig. 7). A detailed dynamical understanding is beyond the scope of this study, and as such future work should continue to investigate how upwelling anomalies drive changes in the nearshore heat budget through modification of the slope Burger number and vertical location of the cross-shelf return flow.

### Climate change effects and implications

Previous work using a multi-model ensemble of global climate forecasts skillfully forecasted longer-duration MHWs up to a year in advance in response to the state of large-scale climate modes such as ENSO^41^. However, the results were variable across different regions and limited to longer duration MHWs due to the monthly resolution of seasonal forecasts. Accurately predicting short-lived MHWs and MCSs across different regions will likely require consideration of region-specific drivers, such as upwelling in EBUS, to supplement global forecasts^41^. Moreover, reliable forecasts of MHWs and MCSs in EBUS will be sensitive to future changes in the coupled ocean–atmosphere system due to anthropogenic climate change, the uncertainties of which remain substantial^42^. There is general agreement that strong El Niño and La Niña events are expected to increase in frequency and intensity over the next few decades, potentially leading to longer-lived MHWs and diminishing MCSs^43^. While the PDO is expected to weaken in intensity and increase in frequency, there is also an expected increase in the phase occurrence of the warm states of the PDO and ENSO, potentially leading to further increases in MHWs and decreases in MCSs^44–46^. Wind-driven upwelling predictions and subsequent impacts in EBUS are among the most uncertain^15,22^. Increases in upwelling-favorable winds (e.g., Bakun hypothesis) could maintain nearshore regions of EBUS as thermal refugia but increases in upper-ocean warming and stratification could reduce the cooling effects of upwelling, leading to competing impacts for nearshore extreme events and large uncertainties in how the frequency and severity of MHWs and MCSs will change^22^. Moreover, as noted by others, the details of how changes in upwelling intensity could modulate warming rates is uncertain, but the prevalence of upwelling (whether intensifying or not) appears sufficient to influence SST trends in EBUS^19,42^. Nonetheless, changes in upwelling and upper-ocean stratification, and the subsequent impact to the nearshore heat budget, are likely to determine whether nearshore ecosystems along EBUS will continue to serve as thermal refugia in a changing climate^9,17,18^.

The nearshore regions of EBUS contribute disproportionately to global ocean productivity and understanding drivers of ecosystem change remains of paramount importance to protecting and conserving these critical habitats^42^. Extreme events (MHWs/MCSs) can drive the decline of ecosystem engineers like giant kelp forests, drive range expansion of species, and lead to changes in primary productivity^15,47–49^. These events can also lead to multi-stressor systems whereby extreme temperature anomalies co-exist with biogeochemical extremes due to conditions (e.g., harmful algal blooms, eutrophication, upwelling and stratification) that can exacerbate coastal ocean acidification and hypoxia (OAH) risk in nearshore habitats^50,51^. Despite the threat to valuable ecosystems, there are limited studies of the drivers of extreme events in the very shallow nearshore region of EBUS^28^. Moreover, it is expected that MHW/MCS exposure in different nearshore environments is likely to be highly variable due to the presence of marine microclimates, where variations in oceanographic conditions and species response to extreme events can be strikingly different over just a few kilometers^33,50,51^. Collectively, this study highlights the critical role that both basin-scale climate modes and regional upwelling play in predicting extreme events and shaping nearshore resilience in EBUS.

## Methods

### Site description

**Figure 8:**
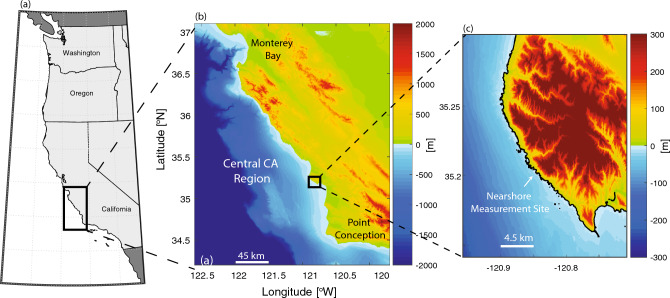
(**a**) US West Coast with Central California region (black box), (**b**) bathymetry and topography of Central California region with nearshore study site location (black box), and (**c**) nearshore measurement site. Maps were generated using MATLAB Version R2022a (https://www.mathworks.com/).

Temperature data were obtained from a long-term, subtidal site located along the central California coast (USA) in the CCS (Fig. 8). Coastal upwelling seasonality dominates the physical and biogeochemical variability in this region, with peak upwelling in the spring (April and May) and moderate upwelling throughout the summer^29,35,52^. This region is also home to two major commercial fishing ports and economically important fisheries, several marine protected areas, and giant kelp forests that support high biodiversity^36^. Data were collected in a shallow (~ 3 m nominal depth) nearshore site adjacent to the Diablo Canyon nuclear power plant (35.2055°N, 120.8500°W; Fig. 8). Originally established as a control site outside the influence of the power plant’s thermal outfall, these data span from 1978–2020 and make up the longest known in-situ temperature dataset in this region. Temperature was measured using thermistors (Table S1) sampling at 20-min intervals.

### MHW and MCS detection and classification

A daily averaged temperature time series was computed and gaps less than two days in length were linearly interpolated^26^. MHWs and MCSs were detected according to the definitions detailed by Hobday et al. and Schlegel et al., respectively, using the MATLAB toolbox from Zhao and Marin^6,53,54^. The climatology, 10th percentile, and 90th percentile for each day of the year were generated using an 11-day moving average. A MHW (MCS) is detected when the daily averaged temperature exceeds (falls below) the 90th (10th) percentile threshold for at least 5 days. If there were multiple MHWs or MCSs that took place with less than 2 days in between, these were combined into a single event. This methodology captures discrete and prolonged extreme temperature events while accounting for seasonal variability. We refer the reader to Hobday et al. and Schlegel et al. for further details^6,53^.

MHW and MCS metrics were first calculated on a per-event basis. The metrics studied were duration, mean intensity, maximum intensity, and cumulative intensity. Intensity is defined as the difference between the daily averaged temperature and the climatology. The cumulative intensity is defined as the integral of intensity over the duration of the event. For MCSs, we refer to the magnitude of the intensity values, which denote negative differences between the daily averaged temperature and the climatology. Both MHWs and MCSs were further categorized following Hobday et al. based on the respective maximum intensity above (MHW) or below (MCS) the respective percentile (90th for MHWs and 10th for MCSs) as moderate (1–2x respective percentile), strong (2–3x), severe (3–4x), and extreme (4–5x)^55^. There were no severe or extreme MHWs or MCSs identified in this study. To investigate interannual variability, we calculated the following metrics on an annual basis: frequency (events per year), event days per year, and yearly cumulative intensity. We also calculated statistics separated into the following groups for both MHWs and MCSs, respectively: all events, moderate events only, and strong events only (Table 1).

### Environmental data

To quantify the role of regional-scale variability on MHWs and MCSs, we utilized the Coastal Upwelling Transport Index (CUTI). The CUTI captures the rate of vertical transport resulting from wind-stress driven Ekman transport and pressure-gradient driven cross-shore geostrophic transport at a 1° latitude resolution along the US West Coast using ocean state estimates and surface wind forcing from several reanalysis products^56^. We obtained daily CUTI values at 35° N from the available time range (1988–2020). A CUTI climatology was then calculated using the same 11-day moving average as the temperature climatology, and deviations from this climatology on each respective day of the year were used to define the CUTI anomaly (CUTIa). Since the calculated CUTI climatology was always positive, a positive CUTIa represents stronger than normal upwelling, while a negative CUTIa denotes either weaker than normal upwelling or downwelling.

We examined two dominant modes of variability in the Pacific: the Pacific Decadal Oscillation (PDO) and the El Niño Southern Oscillation (ENSO). The PDO Index is the monthly amplitude time series of the principal empirical orthogonal function (EOF) of SST anomalies in the North Pacific^57^. We obtained PDO Index data from 1978–2020 (https://www.ncei.noaa.gov/pub/data/cmb/ersst/v5/index/ersst.v5.pdo.dat). Along the CCS, positive PDO values (warm phase) typically correspond with warmer conditions, while negative PDO values (cold phase) correspond with colder conditions^57^. The multivariate ENSO Index (MEI) was used to quantify El Niño/La Niña events from 1979 to 2020 (https://psl.noaa.gov/enso/mei/data/meiv2.data). The MEI (MEI.v2 used here) is the amplitude time series of the principal combined empirical orthogonal function of five different oceanographic and atmospheric variables over the tropical Pacific basin, including SST, sea level pressure, surface zonal and meridional winds, and outgoing longwave radiation^58^. El Niño events (MEI > 0.5) are linked with warm SST anomalies in the CCS and La Niña events (MEI < -0.5) with cold SST anomalies^59^. We note that the PDO is a statistical mode of variability resulting from different physical processes, including teleconnections from ENSO variability in the tropical Pacific, and as such the PDO and MEI are possibly dependent^60^. However, given that other processes contribute to both the PDO and MEI, consideration of the relative sign of these two modes of variability is justified. Likewise, the PDO and MEI are linked with changes in coastal upwelling^61^. For each MHW and MCS, adjusted PDO and MEI values were calculated. Since the PDO and MEI are monthly indices, each MHW and MCS was assigned an average (adjusted) PDO and MEI value weighted by the number of days in each month for each respective MHW and MCS.

### Supplementary Information

Supplementary Table S1.

## Data Availability

Temperature data used here will be made available upon reasonable request from the corresponding author.
