# Author Correction: Effects of basin-scale climate modes and upwelling on nearshore marine heatwaves and cold spells in the California Current

**DOI:** 10.1038/s41598-023-42864-x

**Published:** 2023-09-22

**Authors:** Michael Dalsin, Ryan K. Walter, Piero L. F. Mazzini

**Affiliations:** 1 Physics Department, California Polytechnic State University, San Luis Obispo, CA USA; 2 Virginia Institute of Marine Science, William & Mary, Gloucester Point, VA USA

Correction to: *Scientific Reports* 10.1038/s41598-023-39193-4, published online 31 July 2023

The original version of this Article contained an error in the Methods section, under the subheading ‘Site description’, where the coordinates of the temperature measurement location were incorrect.

“Data were collected in a shallow (~ 3 m nominal depth) nearshore site adjacent to the Diablo Canyon nuclear power plant (35.2277°N, 120.8762°W; Fig. 8).”

now reads:

“Data were collected in a shallow (~ 3 m nominal depth) nearshore site adjacent to the Diablo Canyon nuclear power plant (35.2055°N, 120.8500°W; Fig. 8).”

The original Article has been corrected.

