# Supplementary material for: Effects of basin-scale climate modes and upwelling on nearshore marine heatwaves and cold spells in the California Current
Source: Sci Rep. 2023 Jul 31;13:12389. doi: 10.1038/s41598-023-39193-4 (PMC10390473; doi:10.1038/s41598-023-39193-4)
Supplement: Supplementary file 1 — Supplementary Table S1. [file 41598_2023_39193_MOESM1_ESM.docx]

**Effects of basin-scale climate modes and upwelling on nearshore marine heatwaves and cold spells in the California Current**

**Supplemental Materials**

**Table S1.** Summary of temperature sensors used for the Diablo Canyon data.

| **Manufacturer** | **Dates Used** | **Unit Type** | **Reported Accuracy** | **Reported Resolution** |
| --- | --- | --- | --- | --- |
| ENDECO | 1977–1982 | Type 109 | 0.2°C | 0.1°C |
| Sea Data | 1982–1999 | TR-2 | 0.1°C | 0.01°C |
| Hugrun* | 1999–2016 | Seamon-mini™ | 0.025°C | 0.001°C |
| Star Oddi | 2014–present | Starmon-mini | 0.025°C | 0.001°C |
